# Epigenetic Activation of the KDM6B‐DKK1 Axis Promotes Colorectal Cancer Peritoneal Metastasis Through Wnt/β‐Catenin‐Associated EMT

**DOI:** 10.1111/jcmm.71326

**Published:** 2026-08-23

**Authors:** Zhichao Zhai, Jiajia Chen, Yong Yang, Ming Li

**Affiliations:** ^1^ Tianjin Nankai Hospital Tianjin Medical University Tianjin China; ^2^ Tianjin Key Laboratory of Acute Abdomen Disease Associated Organ Injury and ITCWM Repair Tianjin China; ^3^ Institute of Integrative Medicine for Acute Abdominal Diseases Tianjin China; ^4^ Department of Gastrointestinal Surgery Peking University Shougang Hospital Beijing China

**Keywords:** colorectal cancer, DKK1, epigenetic regulation, epithelial‐mesenchymal transition, KDM6B, peritoneal metastasis, Wnt/β‐catenin

## Abstract

Peritoneal metastasis (PM) is a highly lethal pattern of progression in colorectal cancer (CRC), yet the specific molecular drivers and upstream regulatory mechanisms underlying this process remain incompletely understood. In the present study, public transcriptomic datasets from TCGA and GEO were integrated to identify PM‐associated genes in CRC, and prognostic relevance was evaluated using Kaplan–Meier survival analysis and Cox regression. Stable knockdown and overexpression models were established to investigate the role of DKK1 in CRC cell migration, invasion and peritoneal dissemination using Transwell assays and a mouse peritoneal metastasis model. Multi‐cohort analysis identified 86 genes specifically upregulated in PM, among which DKK1 showed the most consistent overexpression and was significantly associated with poor prognosis in patients with CRC. Functionally, DKK1 knockdown significantly suppressed CRC cell migration, invasion and peritoneal metastatic colonization in vivo, whereas DKK1 overexpression enhanced these malignant phenotypes. Mechanistically, DKK1 promoted metastasis in association with activation of the Wnt/β‐catenin signalling programme and induction of epithelial‐mesenchymal transition. Upstream analysis demonstrated that the histone demethylase KDM6B was positively correlated with DKK1 expression and directly activated DKK1 transcription by binding to its promoter and reducing H3K27me3 enrichment. Rescue experiments further indicated that the pro‐metastatic and epithelial‐mesenchymal transition‐promoting effects of KDM6B were largely dependent on DKK1. Collectively, these findings identify DKK1 as a key driver of CRC PM and establish the KDM6B‐DKK1‐Wnt/β‐catenin‐epithelial‐mesenchymal transition axis as a critical regulatory pathway in this process.

## 1 Introduction

Colorectal cancer (CRC) ranks among the most prevalent and lethal malignancies worldwide, with mortality primarily attributable to metastasis rather than the primary tumour itself [1]. Although the liver and lungs are the most common sites of distant metastasis, peritoneal metastasis (PM) has emerged as a major clinical challenge due to its insidious onset, limited therapeutic options, and extremely poor prognosis [2, 3]. Unlike hematogenous metastases to the liver or lungs, PM typically develops through tumour cell shedding, intra‐abdominal dissemination, and peritoneal implantation [4]. However, the biological underpinnings and molecular regulatory mechanisms of PM remain incompletely elucidated.

Growing evidence suggests that PM is not merely an extension of other metastatic routes but possesses distinct molecular characteristics and evolutionary trajectories [5, 6]. Nevertheless, most studies on CRC PM have focused on clinical risk factors or single‐cohort analyses, lacking cross‐cohort integration and multi‐level validation [7]. Consequently, the identification of key drivers remains limited [8]. Therefore, systematically deciphering the specific molecular landscape of PM and identifying robust, reproducible driver genes is crucial for understanding its pathogenesis and discovering potential therapeutic targets.

Dickkopf‐1 (DKK1) is a key regulator of the canonical Wnt/β‐catenin signalling pathway and has been implicated in various tumour processes including proliferation, invasion, immune evasion, and drug resistance [9, 10, 11, 12]. However, the role of DKK1 in CRC remains controversial, likely depending on tumour type and microenvironmental context. In particular, its expression pattern, functional significance, and upstream regulatory mechanisms in CRC PM have not been systematically investigated. Furthermore, epigenetic regulation is recognized as a fundamental basis of tumour metastasis plasticity. The histone H3K27me3 demethylase KDM6B (JMJD3) has been shown to participate in EMT, invasion, and metastasis‐related transcriptional programmes in multiple cancers [13, 14, 15]. Yet, whether KDM6B drives CRC PM through specific target genes, and whether it forms a functional axis with DKK1, remains unexplored.

In this study, we employed multi‐cohort integrative analysis to delineate the molecular profile of CRC PM and identified DKK1 as a specific and prognostically significant key gene. Through a combination of in vitro and in vivo functional assays, pathway analysis, and epigenetic investigations, we further uncovered the pivotal role of the KDM6B–DKK1–Wnt/EMT axis in driving CRC peritoneal metastasis, thereby providing new theoretical insights for molecular subtyping and potential therapeutic strategies against PM.

## 2 Materials and Methods

### 2.1 Data Acquisition and Processing

Transcriptomic data related to colorectal cancer (CRC) were systematically retrieved from The Cancer Genome Atlas (TCGA) and Gene Expression Omnibus (GEO) databases. The inclusion criteria were as follows: (1) datasets containing at least one of the following tissue types: normal colorectal mucosa, primary CRC tumour, or peritoneal metastasis (PM); (2) a sample size of at least 5 cases; and (3) complete clinical follow‐up information for datasets used in survival analysis. After screening, a total of nine publicly available datasets were included: TCGA Colon Adenocarcinoma/Rectal Adenocarcinoma (TCGA‐COAD/READ), GSE225182 [16], GSE147971 [17], GSE41568 [18], GSE68468 [19], GSE26571 [20], GSE3964 [21], GSE6988 [22], and GSE62321 [23] (Table S1). GEO microarray datasets were background‐corrected and quantile‐normalized using the limma package (v3.54.2), and batch effects were removed using the sva package in R (v4.2.3). The processed datasets were then merged into a unified expression matrix for subsequent analyses [24].

### 2.2 Differential Expression Gene Analysis

Based on the batch‐corrected expression matrix, differential expression analysis was performed using the limma package (v3.54.2) to compare each metastatic site (peritoneum, liver, lung, and lymph node) with primary tumour tissues. Differentially expressed genes (DEGs) were identified using the criteria of |log2 fold change| > 1 and false discovery rate (FDR) < 0.05. Upregulated genes in each metastatic site relative to primary tumours were extracted, and Venn diagrams were generated using the VennDiagram package (v1.7.3) to identify PM‐specific upregulated genes [25]. To further assess the expression stability of candidate genes, expression values from the GSE147971 and GSE41568 datasets were extracted and visualized using the pheatmap package (v1.0.12).

### 2.3 Identification of Prognosis‐Related Genes

In the TCGA‐COAD/READ cohort (*n* = 595), a univariate Cox proportional hazards regression model was applied to assess the correlation between PM‐specific upregulated genes and overall survival (OS). Genes with *p* < 0.05 were considered to have prognostic value. The intersection of prognosis‐related genes and PM‐specific upregulated genes yielded key candidate genes. Patients were stratified into high‐ and low‐expression groups based on the median expression level of the candidate gene, and Kaplan–Meier survival curves were generated. Group differences were compared using the log‐rank test, and hazard ratios (HR) with 95% confidence intervals (CI) were calculated.

### 2.4 Gene Set Enrichment Analysis

For the three PM‐related datasets (GSE225182, GSE147971, and GSE41568), downloaded expression matrices were log_2_‐transformed and quantile‐normalized when necessary. Samples were then divided into high‐ and low‐expression groups based on the median DKK1 expression level. Gene set enrichment analysis (GSEA v4.3.2) was performed using the Hallmark gene sets (v2023.2) from the MSigDB database to evaluate pathway enrichment in DKK1‐high samples [26]. Significance was assessed using the normalized enrichment score (NES) and FDR values (FDR < 0.25 was considered significantly enriched).

### 2.5 Cell Lines and Culture Conditions

All CRC cell lines used in this study were obtained from the Shanghai Cell Bank of the Chinese Academy of Sciences, including: HT29 (SCSP‐5032), Caco‐2 (SCSP‐5027), HCT116 (SCSP‐5076), SW620 (SCSP‐5234), LoVo (SCSP‐514), Colo320 (TCHu 14), and SW48 (SCSP‐5033). HCT116, SW620, and LoVo cells were cultured in RPMI‐1640 (Gibco, cat. 11875093), whereas HT29, Caco‐2, SW48, and Colo320 cells were maintained in DMEM (Gibco, cat. 11995065) supplemented with 10% foetal bovine serum (FBS; Gibco, cat. A3160802), 100 U/mL penicillin, and 100 μg/mL streptomycin (Gibco, cat. 15140122) and maintained at 37°C in a humidified atmosphere containing 5% CO_2_. All cell lines were authenticated by short tandem repeat (STR) analysis and regularly screened for mycoplasma contamination.

### 2.6 Stable Knockdown and Overexpression of DKK1

Two specific shRNA sequences targeting human DKK1 (RefSeq: NM_012242.4) were designed: shDKK1#1 (5′‐CCGGAAGGTCTGTCTTGCCGGATTTCAAGAGAATCCGGCAAGACAGACCTTTTTTTG‐3′) and shDKK1#2 (5′‐CCGGAAGCTTTGGTAATGATCATTCAAGAGAATGATCATTACCAAAGCTTTTTTTG‐3′). A scrambled shRNA sequence (sh‐scramble: 5′‐ACCTCGGATAGTGCGGGTATTGTGATTCAAGAGATCACAATACCCGCACTATCCTT‐3′) was used as the negative control. All shRNA sequences were cloned into the pLKO.1‐puro vector (Addgene, plasmid #8453). Lentiviral packaging was performed using the psPAX2 (Addgene, #12260) and pMD2.G (Addgene, #12259) helper plasmids by transfecting 293 T cells (ATCC CRL‐3216) with Lipofectamine 3000 (Thermo Fisher, cat# L3000015). Virus‐containing supernatants were used to infect HCT116 and SW620 cells, followed by selection with 2 μg/mL puromycin (Gibco, cat# A1113803) for 7 days to establish stable knockdown cell lines. For DKK1 overexpression, full‐length DKK1 cDNA was cloned into the pLVX‐puro vector (Takara Bio, cat# 632164). Lentiviral infection of Caco‐2 and SW48 cells followed by puromycin selection yielded stable overexpression cell lines. For KDM6B knockdown, two shRNA sequences were used: shKDM6B#1 (5′‐CCGGGCCTCATCCTTCCAGGAGTCTCGAGACTCCTGGAAGGATGAGGCTTTTTG‐3′) and shKDM6B#2 (5′‐CCGGGGAATGTGACTTCAAATAACTCGAGTTATTTGAAGTCACATTCCCTTTTTG‐3′). For in vitro rescue experiments, KDM6B overexpression was achieved by transient transfection of a KDM6B‐pcDNA3.1‐Flag plasmid using Lipofectamine 3000 according to the manufacturer's instructions.

### 2.7 Quantitative Real‐Time PCR

Total RNA was extracted using TRIzol reagent (Invitrogen, cat. 15596026), and RNA concentration and purity were determined using a NanoDrop 2000 spectrophotometer (Thermo Scientific). Reverse transcription was performed with the PrimeScript RT Reagent Kit with gDNA Eraser (Takara Bio, cat. RR047A). Quantitative PCR was carried out using TB Green Premix Ex Taq II (Takara Bio, cat. RR820A) on an ABI 7500 Fast Real‐Time PCR System (Applied Biosystems). Thermal cycling conditions were: 95°C for 30 s, followed by 40 cycles of 95°C for 5 s and 60°C for 34 s. Primer sequences were as follows: DKK1‐F (5′—ATAGCACCTTGGATGGGTATTCC—3′), DKK1‐R (5′—CTGATGACCGGAGACAAACAG—3′), KDM6B‐F (5′—CCAGCCCGGCCAGCCTGCTCAAA‐3′), KDM6B‐R (5′—GAGGTCGTGCCTCCGGTTCC‐3′), GAPDH‐F (5′‐CAGGAGGCATTGCTGATGAT‐3′), GAPDH‐R (5′‐GAAGGCTGGGGCTCATTT‐3′). GAPDH served as the internal reference gene, and relative expression levels were calculated using the 2^−ΔΔCt^ method. All experiments were independently performed in triplicate.

### 2.8 Western Blotting

Clinical tissue samples (normal colorectal mucosa, primary CRC tumour, and PM, *n* = 3 each) were collected with informed patient consent and institutional ethical approval. Cell samples included HCT116, SW620, Caco‐2, and SW48 cells under various treatments. Total protein was extracted using RIPA lysis buffer (Beyotime, cat P0013B), and protein concentration was determined using the BCA method (Pierce, cat 23,225). Equal amounts of protein (20 μg) were separated by 10% or 12% SDS‐PAGE and transferred to 0.45 μm PVDF membranes (Millipore, cat IPVH00010). Membranes were blocked with 5% non‐fat milk for 1 h at room temperature and then incubated with primary antibodies overnight at 4°C. Primary antibodies included: anti‐DKK1 (Abcam, cat ab109416, 1:1000), anti‐KDM6B (Cell Signalling Technology, cat 33510, 1:1000), anti‐E‐cadherin (CST, cat 3195, 1:1000), anti‐N‐cadherin (CST, cat 13116, 1:1000), anti‐Vimentin (CST, cat 5741, 1:1000), anti‐Slug (CST, cat 9585, 1:1000), anti‐Snail1 (CST, cat 3879, 1:1000), anti‐Twist1 (CST, cat 46702, 1:1000), anti‐MMP9 (CST, cat 13667, 1:1000), anti‐H3K27me3 (CST, cat 9733, 1:1000), anti‐H3 (CST, cat 4499, 1:1000), and anti‐GAPDH (CST, cat 5174, 1:5000). After washing, membranes were incubated with HRP‐conjugated secondary antibody (CST, cat 7074, 1:5000) for 1 h at room temperature. Protein bands were visualized using Clarity Western ECL Substrate (Bio‐Rad, cat 1705060) on a ChemiDoc MP Imaging System (Bio‐Rad). Western blot band intensities were quantified using ImageJ software. Protein levels were normalized to GAPDH, whereas H3K27me3 levels were normalized to total H3, and results were presented as fold change relative to the control group.

### 2.9 Immunohistochemistry

Formalin‐fixed, paraffin‐embedded tissue sections were deparaffinized, rehydrated, and subjected to antigen retrieval in citrate buffer (pH 6.0) at 95°C for 15 min. Endogenous peroxidase activity was blocked with 3% H_2_O_2_ for 10 min at room temperature. After blocking with 10% goat serum for 30 min, sections were incubated with anti‐DKK1 primary antibody (Abcam, cat ab109416, 1:200) overnight at 4°C. The following day, sections were incubated with a biotin‐labelled secondary antibody (ZSGB‐BIO, cat PV‐6000) for 30 min at room temperature, followed by colour development with a DAB substrate kit (ZSGB‐BIO, cat ZLI‐9018). Sections were counterstained with haematoxylin, dehydrated, and mounted. Staining results were independently evaluated by two experienced pathologists under blinded conditions.

### 2.10 Cell Migration and Invasion Assays

Cell migration was assessed using 24‐well Transwell inserts (Corning, cat. 3422, 8 μm pore size). A total of 5 × 10^4^ cells resuspended in 200 μL serum‐free medium were seeded into the upper chamber; the lower chamber contained 600 μL complete medium with 10% FBS as a chemoattractant. For invasion assays, Transwell inserts (Corning, cat. 354480) were pre‐coated with Matrigel (Corning, cat 356234), and 1 × 10^5^ cells were seeded per well. Cells were incubated at 37°C under 5% CO_2_ for 24 h (migration) or 48 h (invasion). After incubation, non‐migrated/non‐invaded cells on the upper surface were removed with a cotton swab. Cells that had migrated/invaded through the membrane to the lower surface were fixed with 4% paraformaldehyde for 15 min and stained with 0.1% crystal violet for 20 min. Five random fields (200× magnification) were photographed under an inverted microscope (Olympus IX73), and the number of transmigrated cells was counted. All experiments were performed in triplicate.

### 2.11 Mouse Peritoneal Metastasis Model

Female BALB/c nude mice (6–8 weeks old, Beijing Vital River Laboratory Animal Technology Co. Ltd.) were housed under specific pathogen‐free (SPF) conditions (temperature 22°C ± 2°C, humidity 50% ± 10%, 12 h light/dark cycle). All animal experiments were approved by the Animal Ethics Committee of Peking University (Approval No. LA2020404). Cells in logarithmic growth phase were trypsinized, washed twice with PBS, and resuspended in sterile PBS at a density of 1 × 10^6^ cells/100 μL. Experimental groups were as follows: DKK1 knockdown experiment with Scramble, shDKK1‐#1, and shDKK1‐#2 groups in HCT116 and SW620 cells, and DKK1 overexpression experiment with Control and DKK1‐OV groups in Caco‐2 and SW48 cells. Each group consisted of 6–8 mice. Mice were intraperitoneally injected with 100 μL cell suspension and monitored regularly. After 4–6 weeks, mice were euthanized, and peritoneal organs were carefully removed. Visible metastatic nodules (> 1 mm in diameter) on the peritoneal surface and mesentery were counted, and each nodule was histologically confirmed.

### 2.12 In Vivo Imaging Analysis

Cells stably expressing luciferase (via lentiviral transduction of pGL4.51[luc2/CMV/Neo] vector, Promega, cat. E1320) were further engineered to generate the indicated stable cell lines for rescue experiments, including Vector + shNC, shDKK1 + Vector, shDKK1 + KDM6B‐OE, DKK1‐OE + Vector, and DKK1‐OE + shKDM6B. Cells (2 × 10^6^ per mouse) were intraperitoneally injected into 6–8‐week‐old BALB/c nude mice (*n* = 6 per group). On Day 14 after injection, mice were intraperitoneally injected with D‐luciferin potassium salt (150 mg/kg, PerkinElmer, cat. 122799). After 10 min, bioluminescence signals were detected using an IVIS Spectrum imaging system (PerkinElmer). Total photon flux (photons/s/cm^2^/sr) was quantified using Living Image 4.5 software (PerkinElmer). Mice were euthanized on Day 21, and peritoneal nodules were collected for haematoxylin and eosin (H&E) staining.

### 2.13 Chromatin Immunoprecipitation

A total of 1 × 10^7^ treated HCT116 cells were cross‐linked with 1% formaldehyde for 10 min at room temperature, and the reaction was quenched with 125 mM glycine. Cells were lysed, and chromatin was sheared to fragments of 200–500 bp using a Bioruptor Pico (Diagenode). Ten percent of the sheared chromatin was reserved as the input control. The remaining chromatin was incubated overnight at 4°C with rotation with anti‐KDM6B (CST, cat. 33510), anti‐H3K27me3 (CST, cat. 9733), or rabbit IgG (CST, cat. 2729). Protein A/G magnetic beads were then added and incubated for 2 h at 4°C. Beads were sequentially washed with low‐salt, high‐salt, LiCl, and TE buffers. Immune complexes were eluted in ChIP elution buffer (1% SDS, 0.1 M NaHCO_3_) at 65°C overnight. DNA was purified by proteinase K digestion and phenol‐chloroform extraction, followed by qPCR analysis. qPCR primers covered eight regions (P1–P8, each approximately 200 bp) within the DKK1 promoter, and the primer sequences are provided in Table S2. Relative enrichment was calculated using the 2^−ΔΔCt^ method, with normalization to the IgG control group.

### 2.14 Statistical Analysis

All experiments were performed at least three times independently. Continuous data are presented as mean ± standard deviation. Comparisons between two groups were performed using Student's *t*‐test; multiple group comparisons were conducted using one‐way analysis of variance (ANOVA) with Tukey's post hoc test when appropriate. Survival analysis was performed using the Kaplan–Meier method and log‐rank test. Correlations were analyzed using Pearson's correlation coefficient. Statistical analyses were performed using GraphPad Prism 9.4.1 and R (v4.2.3). A *p*‐value < 0.05 was considered statistically significant.

## 3 Results

### 3.1 Distinct Molecular Features of PM Colorectal Cancer

**FIGURE 1:**
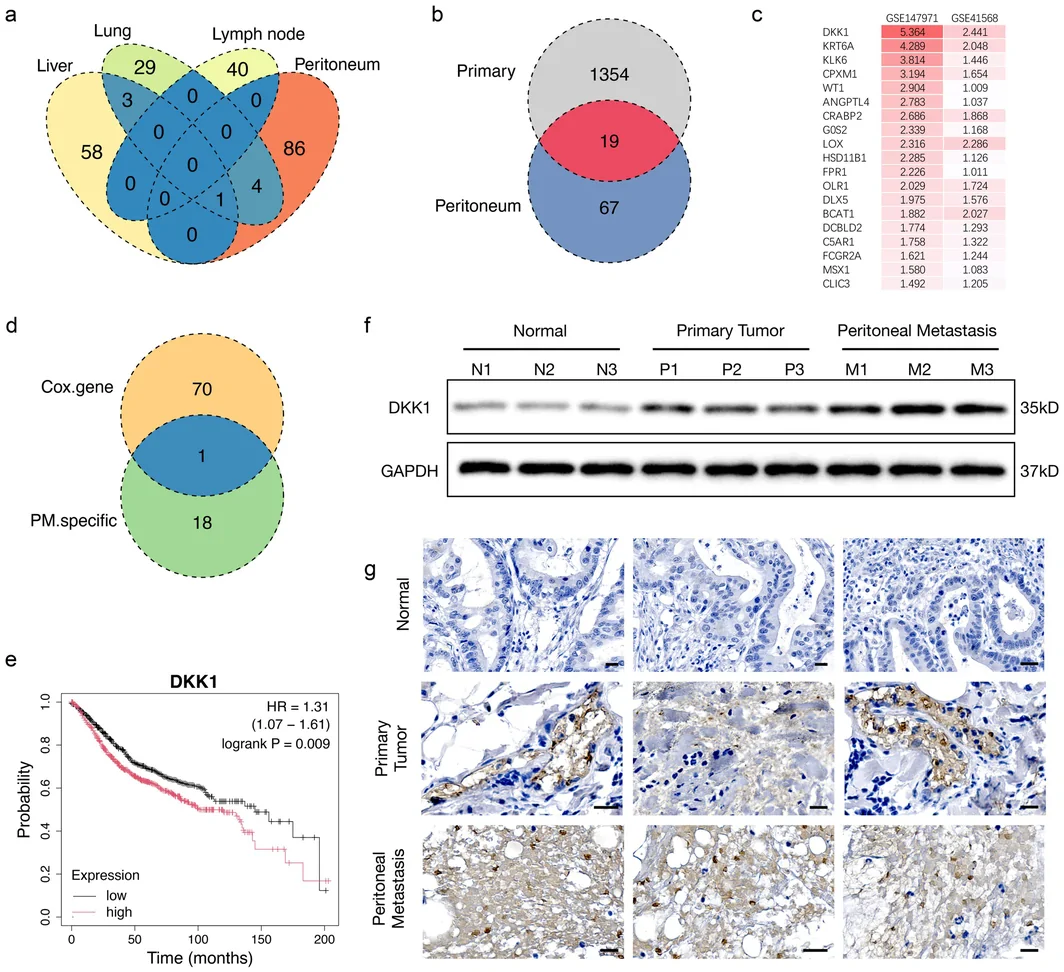
DKK1 is specifically highly expressed in colorectal cancer peritoneal metastasis and predicts poor prognosis. (a) Venn diagram of differentially upregulated genes in different metastatic sites of colorectal cancer (liver, lung, lymph node and peritoneum) compared with primary tumours. Peritoneal metastasis has 86 specifically upregulated genes, suggesting its molecular independence. (b) Overlap of upregulated genes in peritoneal metastasis and primary tumours. A total of 67 peritoneal‐specific upregulated genes were identified, of which only 19 were shared with primary tumours. (c) Heatmap of cross‐validation of peritoneal‐specific genes in two independent GEO datasets (GSE147971 and GSE41568). DKK1 is significantly upregulated in peritoneal metastases across multiple cohorts. (d) Intersection of peritoneal‐specific genes and prognosis‐related genes screened by Cox regression. Only DKK1 simultaneously meets the criteria of peritoneal‐specific expression and prognostic relevance. (e) Kaplan–Meier survival curves of patients with high versus low DKK1 expression in the TCGA cohort. Patients with high DKK1 expression have significantly shorter overall survival (HR = 1.31, 95% CI: 1.07–1.61, log‐rank *p* = 0.009). (f) Western blot detection of DKK1 protein expression in normal tissues (N), primary tumours (P), and peritoneal metastasis (PM) tissues. GAPDH serves as the internal control. (g) Representative images of DKK1 immunohistochemical staining. Scale bar: 50 μm.

To systematically elucidate the molecular basis of PM colorectal cancer, we integrated and analyzed transcriptomic data from multiple independent datasets from TCGA and GEO. By comparing differentially expressed genes between peritoneal, liver, lung, and lymph node metastases relative to primary tumours, we found that peritoneal metastasis harboured 86 specifically upregulated genes, with minimal overlap among other metastatic sites (Figure 1a), suggesting a unique molecular regulatory pattern in PM. Further comparison of differential expression profiles between peritoneal metastasis and primary tumours identified 67 genes specifically upregulated in peritoneal metastasis, of which only 19 were also upregulated in primary tumours (Figure 1b), further confirming the biological distinctiveness of PM.

### 3.2 DKK1 Is a Stably Upregulated Key Prognostic Gene in PM

To verify the reliability of candidate genes, we cross‐validated the peritoneal‐specific genes in two independent GEO datasets (GSE147971 and GSE41568). Heatmap analysis showed that Dickkopf‐1 (DKK1) exhibited the most significant and stable upregulation in peritoneal metastasis tissues across multiple cohorts (Figure 1c). Survival analysis further revealed the clinical significance of DKK1: univariate Cox regression identified 71 genes significantly associated with overall survival in CRC patients; intersecting these with the peritoneal‐specific gene set yielded only one common gene, DKK1 (Figure 1d). Kaplan–Meier survival curves demonstrated that patients with high DKK1 expression had significantly shorter overall survival (hazard ratio HR = 1.31, 95% confidence interval: 1.07–1.61, log‐rank *p* = 0.009) (Figure 1e and Table S3), indicating that DKK1 is not only a characteristic molecule of PM but also a potential poor prognostic marker.

### 3.3 DKK1 Is Highly Expressed in Peritoneal Metastasis Tissues

Western blot analysis showed that DKK1 expression was nearly undetectable in normal colorectal mucosa, moderately elevated in primary tumours, and most markedly and consistently enhanced in peritoneal metastasis tissues (Figure 1f). Immunohistochemistry staining further confirmed this expression pattern: DKK1 was essentially negative in normal mucosa, focally positive in primary tumours, while displaying widespread and intense cytoplasmic staining in peritoneal metastases (Figure 1g). This tissue‐specific expression pattern is highly consistent with the biological function of DKK1 as a driver of PM.

### 3.4 DKK1 Promotes Migration and Invasion of CRC Cells

**FIGURE 2:**
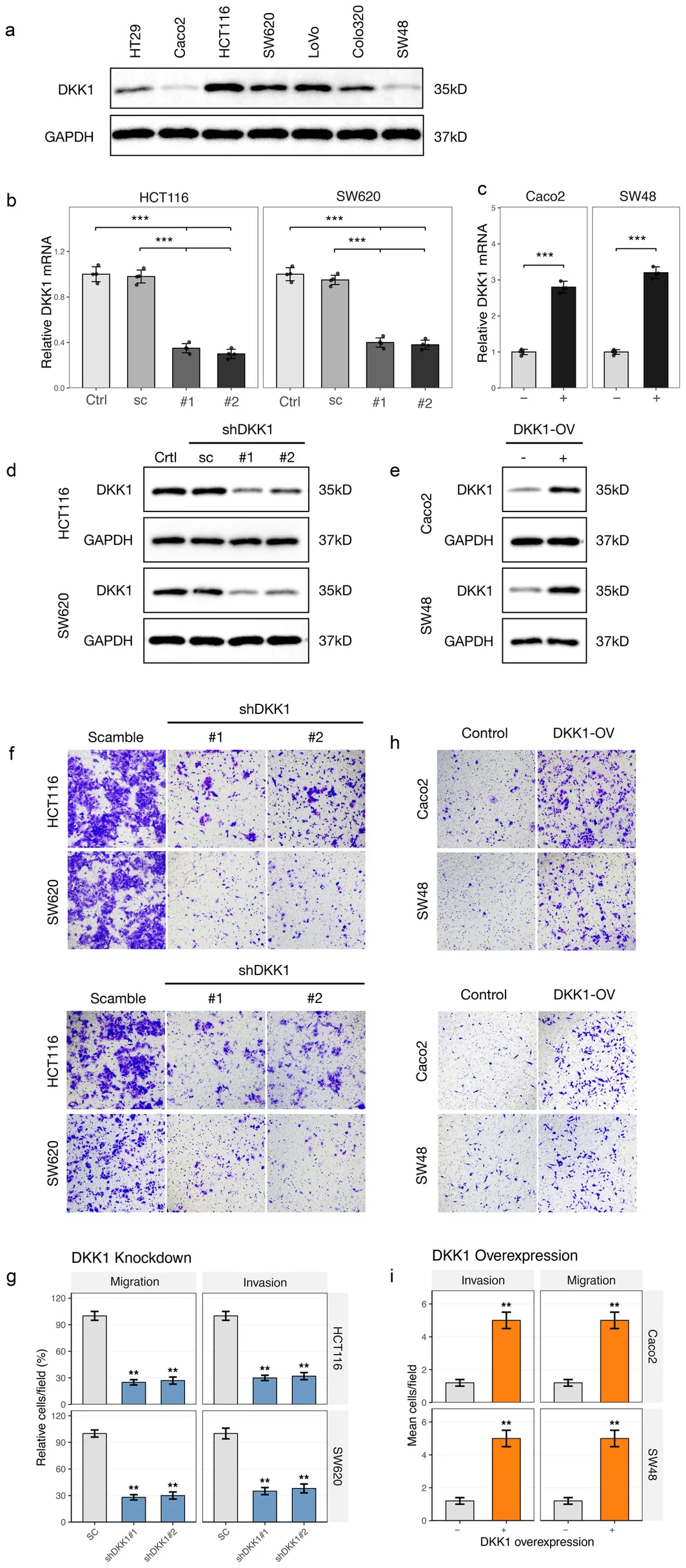
DKK1 promotes migration and invasion of colorectal cancer cells. (a) Western blot detection of basal DKK1 protein expression in seven commonly used colorectal cancer cell lines. (b) RT‐qPCR detection of the knockdown efficiency of shDKK1‐#1 and shDKK1‐#2 on DKK1 mRNA in HCT116 and SW620 cells. (c) RT‐qPCR detection of mRNA expression levels of DKK1 overexpression (DKK1‐OV) in Caco2 and SW48 cells. (d) Western blot validation of DKK1 protein knockdown by shDKK1‐#1 and shDKK1‐#2 in HCT116 and SW620 cells. (e) Western blot validation of successful DKK1 overexpression in Caco2 and SW48 cells. (f, g) Representative images and quantitative analysis of Transwell migration and invasion assays (crystal violet staining) showing that knockdown of DKK1 inhibits migration and invasion of HCT116 and SW620 cells. (h, i) Representative images and quantitative analysis of Transwell migration and invasion assays showing that DKK1 overexpression enhances migration and invasion abilities of Caco_2_ and SW48 cells.

To investigate the biological function of DKK1, we first examined the basal expression levels of DKK1 in seven commonly used CRC cell lines. Western blot results indicated that DKK1 expression was higher in HCT116, SW620, LoVo, and Colo320 cell lines, whereas it was relatively lower in Caco2 and SW48 cell lines (Figure 2a). Based on this expression profile, we selected HCT116 and SW620 cells to establish stable DKK1 knockdown models, and Caco2 and SW48 cells to establish overexpression models. RT‐qPCR and Western blot validation demonstrated that both shDKK1‐#1 and shDKK1‐#2 effectively knocked down DKK1 expression (mRNA knockdown efficiency > 60%, *p* < 0.001) (Figure 2b,d), whereas the overexpression vector increased DKK1 expression by approximately 3‐ to 4‐fold (Figure 2c,e).

Functional assays showed that DKK1 knockdown significantly inhibited the migration and invasion abilities of HCT116 and SW620 cells, reducing the number of transmigrated cells to 25%–35% of the control group (*p* < 0.01) (Figure 2f,g). Conversely, overexpression of DKK1 markedly enhanced the migration and invasion capacities of Caco2 and SW48 cells, increasing the number of transmigrated cells by about 3‐ to 4‐fold (*p* < 0.01) (Figure 2h,i). These results consistently indicate that DKK1 positively regulates the invasive phenotype of CRC cells.

### 3.5 DKK1 Promotes Peritoneal Metastasis Formation In Vivo

**FIGURE 3:**
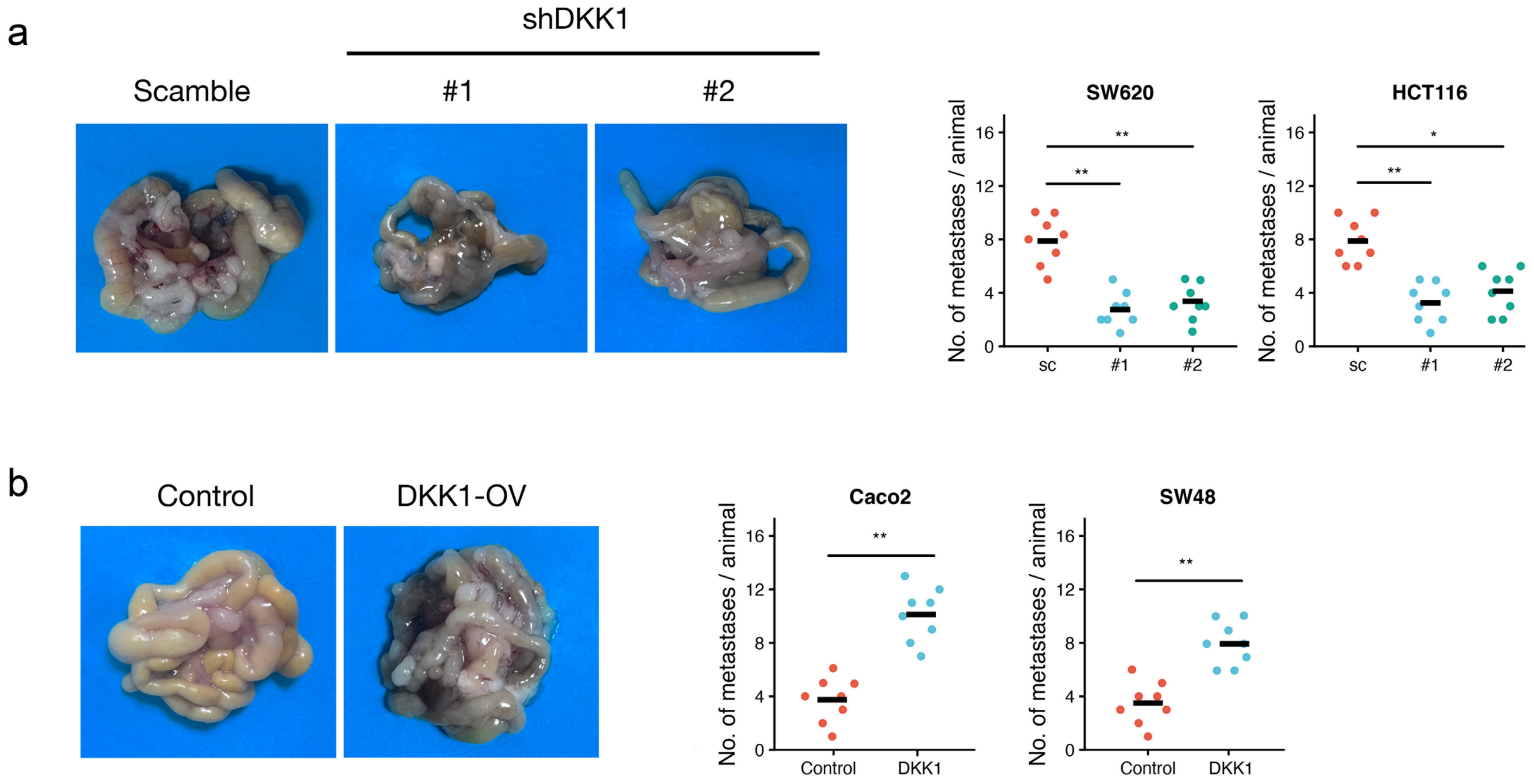
DKK1 promotes peritoneal metastasis of colorectal cancer cells in vivo. (a) DKK1 knockdown inhibits peritoneal metastasis in vivo. Left: Representative images of intraperitoneal metastatic nodules; right: Quantitative statistics of peritoneal metastatic nodule counts (* *p* < 0.05, **p < 0.01). (b) DKK1 overexpression enhances peritoneal metastasis in vivo. Left: Representative images of intraperitoneal metastatic nodules; right: Quantitative statistics of peritoneal metastatic nodule counts (**p < 0.01).

To verify the pro‐metastatic role of DKK1 in vivo, we established a mouse peritoneal metastasis model. Following intraperitoneal injection of HCT116 and SW620 cells stably expressing shDKK1, the number of peritoneal metastatic nodules in the shDKK1 groups was significantly reduced compared with the Scramble control group (Figure 3a). In contrast, Caco2 and SW48 cells overexpressing DKK1 formed more numerous and denser peritoneal metastatic nodules in vivo (Figure 3b). Quantitative analysis confirmed that DKK1 overexpression significantly increased the number of metastatic nodules (*p* < 0.01). These in vivo results are consistent with the in vitro functional experiments, confirming the critical role of DKK1 in driving CRC peritoneal metastasis.

### 3.6 DKK1 Exerts Its Function by Activating the Wnt/β‐Catenin and EMT Pathways

**FIGURE 4:**
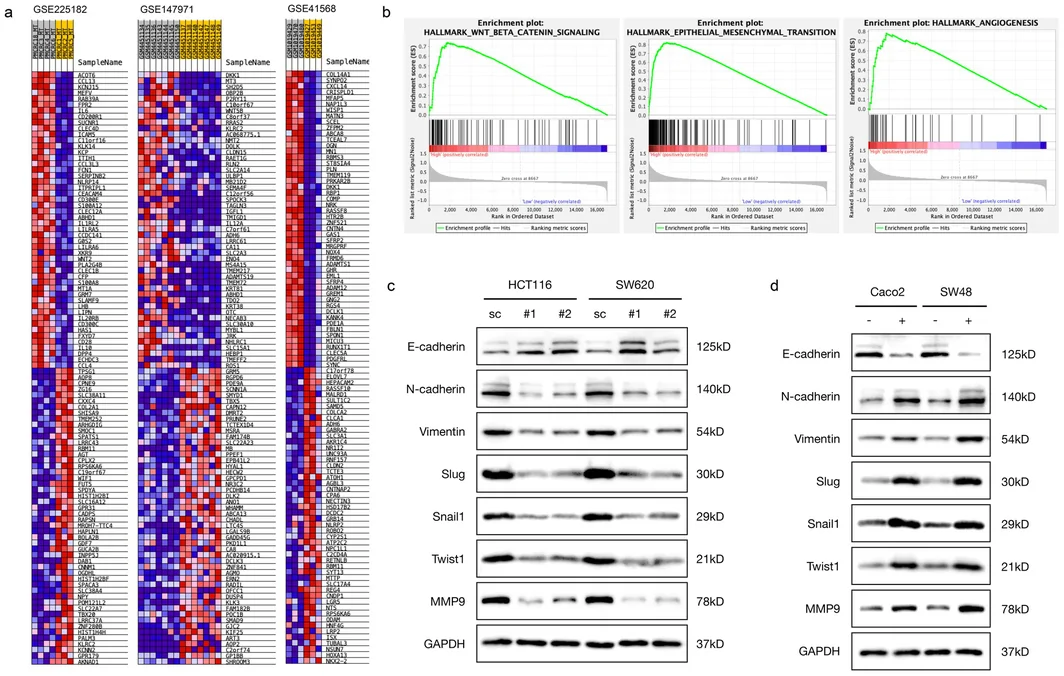
DKK1 promotes an invasive phenotype by activating the Wnt/β‐catenin signalling and EMT pathways. (a) Heatmap of differentially expressed genes based on GSE225182, GSE147971, and GSE41568 datasets, showing significant upregulation of migration‐, EMT‐, and extracellular matrix remodelling‐related genes in DKK1‐high samples. (b) GSEA enrichment analysis shows significant enrichment of Wnt/β‐catenin signalling, EMT, and Angiogenesis pathways in DKK1‐high samples. (c) Western blot analysis of EMT‐related protein expression after DKK1 knockdown in HCT116 and SW620 cells. (d) Western blot analysis of EMT‐related protein expression after DKK1 overexpression in Caco_2_ and SW48 cells.

To elucidate the mechanism of action of DKK1, we performed transcriptomic analysis on three independent CRC cohorts (GSE225182, GSE147971, and GSE41568). Group comparisons based on DKK1 expression levels revealed that genes associated with cell migration, extracellular matrix remodelling, and epithelial‐mesenchymal transition (EMT) were significantly upregulated in DKK1‐high samples (Figure 4a). Gene set enrichment analysis (GSEA) further demonstrated that the Wnt/β‐catenin signalling pathway, EMT pathway, and angiogenesis pathway were significantly enriched in DKK1‐high samples (Figure 4b and Table S4), suggesting that DKK1 may promote metastasis by activating these pro‐metastatic signalling networks.

At the protein level, knockdown of DKK1 led to upregulation of the epithelial marker E‐cadherin, while downregulating the mesenchymal markers N‐cadherin and Vimentin, as well as the EMT transcription factors Slug, Snail1, Twist1, and MMP9 (Figure 4c). Conversely, overexpression of DKK1 inhibited E‐cadherin expression and enhanced the expression of the mesenchymal markers (Figure 4d). These results indicate that DKK1 enhances tumour cell invasion and metastasis by activating Wnt/β‐catenin signalling and inducing the EMT programme.

### 3.7 KDM6B Transcriptionally Regulates DKK1 Expression

**FIGURE 5:**
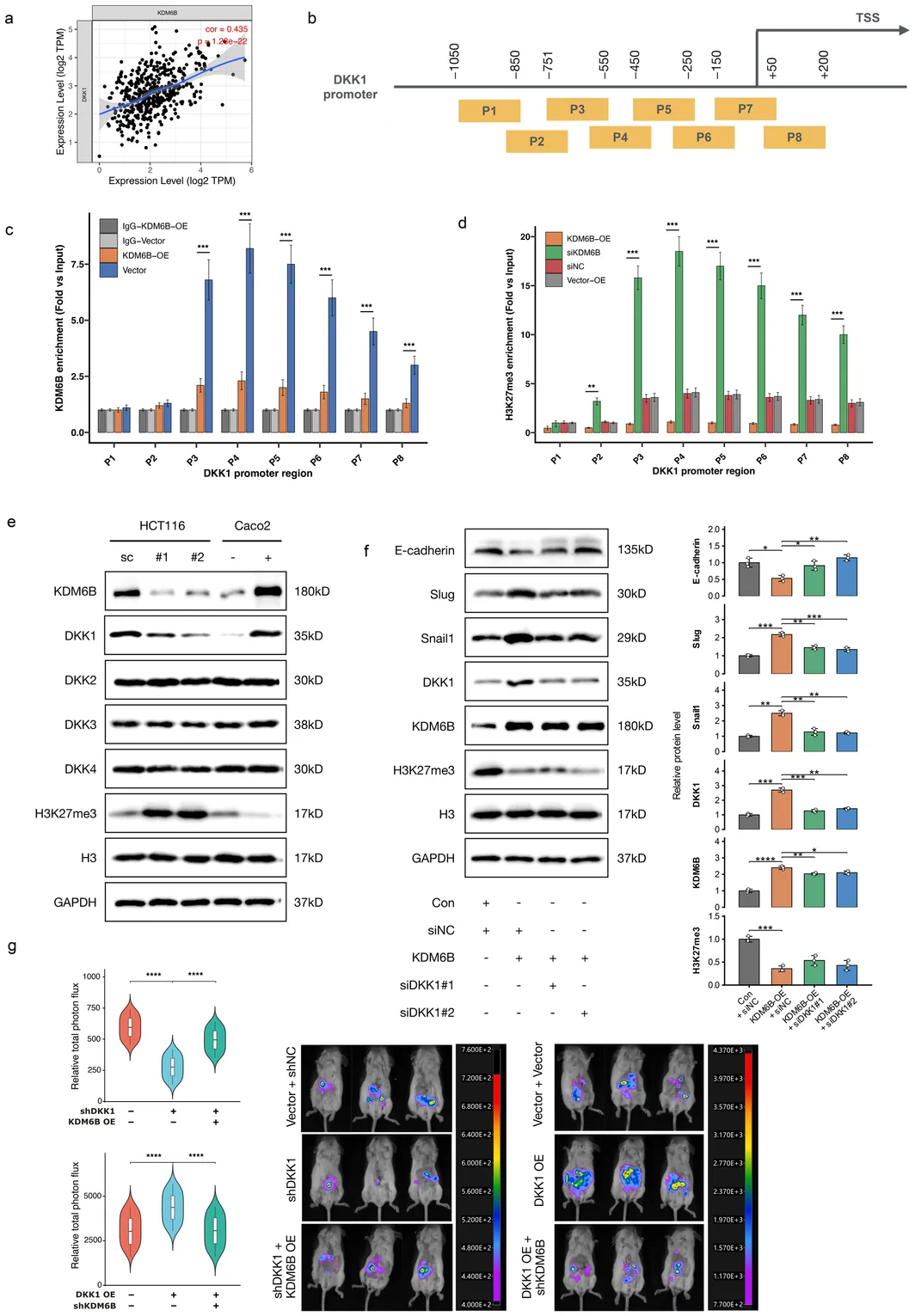
KDM6B drives peritoneal metastasis by epigenetically regulating DKK1 transcription. (a) Scatter plot showing correlation between DKK1 and KDM6B mRNA expression in the TCGA‐COAD cohort (Pearson *r* = 0.435, *p* = 1.24 × 10^−22^). (b) Schematic diagram of the DKK1 gene promoter region, showing the eight ChIP‐qPCR assay fragments (P1–P8). (c) ChIP‐*q*PCR detection of KDM6B binding enrichment in the DKK1 promoter region (*n* = 3). (d) ChIP‐*q*PCR detection of H3K27me3 enrichment levels in the DKK1 promoter region (*n* = 3). (e) Western blot detection of the effects of KDM6B knockdown or overexpression on DKK1 and H3K27me3 expression. (f) Western blot analysis and densitometric quantification of EMT‐related proteins in control cells, KDM6B‐overexpressing cells, and KDM6B‐overexpressing cells with DKK1 knockdown using siDKK1#1 or siDKK1#2. The four lanes correspond to control + siNC, KDM6B overexpression + siNC, KDM6B overexpression + siDKK1#1, and KDM6B overexpression + siDKK1#2, respectively. (g) Representative bioluminescence images and quantitative analysis of total photon flux in the mouse peritoneal metastasis model (*n* = 6).

To explore the upstream regulatory mechanisms of DKK1, we analyzed the correlation between DKK1 and other genes in the TCGA‐COAD cohort. The results showed that DKK1 mRNA expression was significantly positively correlated with the histone demethylase KDM6B (Pearson *r* = 0.435, *p* = 1.24 × 10^−22^) (Figure 5a), suggesting that KDM6B may regulate DKK1 transcription.

Chromatin immunoprecipitation (ChIP)‐qPCR analysis revealed that KDM6B specifically bound to the P3‐P6 region of the DKK1 promoter (from −550 to −51 bp relative to the transcription start site) (Figure 5b,c). Functional experiments demonstrated that knockdown of KDM6B significantly increased H3K27me3 modification levels in the DKK1 promoter region (P3 region: 15.8 ± 1.2 vs. 3.5 ± 0.4, *p* < 0.0001) and suppressed DKK1 expression, whereas overexpression of KDM6B reduced H3K27me3 levels and promoted DKK1 expression (Figure 5d,e). Notably, KDM6B regulation was specific, with no significant effect on other DKK family members (DKK2/3/4).

### 3.8 The KDM6B–DKK1 Axis Drives Peritoneal Metastasis

To determine whether DKK1 mediates KDM6B‐induced EMT, we knocked down DKK1 in KDM6B‐overexpressing cells using two independent siRNAs. This rescue design compared control + siNC cells with KDM6B‐overexpressing cells and with two KDM6B‐overexpressing groups in which DKK1 was depleted by siDKK1#1 or siDKK1#2. KDM6B overexpression increased DKK1, Slug and Snail1 expression and reduced E‐cadherin levels, whereas DKK1 knockdown partially reversed these EMT‐related changes (Figure 5f). These results indicate that KDM6B promotes EMT, at least in part, through DKK1.

In vivo experiments further validated the functional necessity of the KDM6B–DKK1 axis. In the peritoneal metastasis model, knockdown of DKK1 reduced luciferase signal by 58.7% (*p* < 0.0001), while co‐expression of KDM6B restored metastatic capacity (recovery to 92.4%, *p* > 0.05) (Figure 5g). Conversely, overexpression of DKK1 increased the metastatic signal by 3.2‐fold, but this effect was significantly attenuated when KDM6B was co‐knocked down (only 1.3‐fold increase, *p* < 0.0001). These results indicate that KDM6B is an essential mediator for DKK1‐driven peritoneal metastasis and targeting the KDM6B–DKK1 signalling axis may represent a new strategy to block peritoneal metastasis.

## 4 Discussion

Through the integration of multi‐omics data and multi‐level experimental validation, this study systematically reveals that colorectal cancer peritoneal metastasis (PM) possesses molecular features distinct from other metastatic types and, for the first time, elucidates the central role of the KDM6B–DKK1–Wnt/β‐catenin–EMT axis in driving PM. Our research not only provides novel insights into the biological heterogeneity of colorectal cancer metastasis at the fundamental level but also offers potential diagnostic markers and therapeutic targets for this clinically poor‐prognosis metastatic subtype.

Unlike previous studies that predominantly focused on liver and lung metastases, this work employed cross‐cohort integrated analysis of multiple public datasets and demonstrated, at the transcriptomic level, that peritoneal metastasis harbours 86 specifically upregulated genes with minimal overlap with other metastatic sites. This finding supports the notion at a molecular level that PM represents a metastasis mode with distinct biological behaviours, rather than a simple extension of hematogenous or lymphatic metastasis. This provides a theoretical basis for clinically adopting differential treatment strategies for PM (such as intraperitoneal chemotherapy and cytoreductive surgery) and suggests that future research should pay greater attention to the microenvironment specificity of metastatic sites.

Although DKK1 has been extensively studied in various tumours, its role in colorectal cancer remains controversial, particularly in the context of peritoneal metastasis [27, 28, 29]. Through multi‐cohort cross‐validation, this study is the first to establish DKK1 as a key gene that is most stably upregulated in PM and independently associated with patient prognosis. Notably, DKK1 is the only gene among the peritoneal‐specific genes that simultaneously possesses significant prognostic value, which grants it translational potential from a biological marker toward clinical application. We further confirmed at the protein level that DKK1 exhibits sustained high expression in PM tissues, suggesting that it may function not only during metastasis initiation but also in the colonization and growth maintenance of metastatic lesions.

Classical theory holds that DKK1 is an inhibitor of the Wnt/β‐catenin pathway [30]; however, in our colorectal cancer PM model, we observed the opposite effect that high DKK1 expression significantly enriched Wnt/β‐catenin signalling and strongly induced an EMT phenotype. This seemingly contradictory phenomenon aligns with recent research trends indicating that, within the tumour microenvironment, DKK1 may exert pro‐invasive functions through non‐canonical Wnt signalling (such as the Wnt/PCP or Wnt/Ca^2+^ pathway) or microenvironment‐dependent interactions with specific receptors (e.g., the Kremen/LRP6 complex). Our experiments confirmed that DKK1 systematically downregulates E‐cadherin while upregulating N‐cadherin, Vimentin, and key EMT transcription factors (Snail1, Slug, Twist1), thereby providing a clear molecular phenotypic basis for explaining its pro‐metastatic effects.

One of the most significant mechanistic discoveries of this study is the revelation that KDM6B directly activates DKK1 transcription by removing H3K27me3 modifications in the DKK1 promoter region. This regulation exhibits clear site specificity (enriched in the proximal promoter regions P3–P6) and gene specificity (no effect on other DKK family members). More importantly, through in vitro rescue experiments and in vivo functional restoration assays, we demonstrated that KDM6B‐mediated regulation of EMT and peritoneal metastasis strictly depends on DKK1, thereby establishing the KDM6B–DKK1 axis as a functionally complete and causally defined driver of metastasis. This offers a new perspective for understanding how epigenetic modifications influence metastasis specificity by regulating key effector molecules.

From a therapeutic perspective, the KDM6B–DKK1 axis may represent a potential target for CRC peritoneal metastasis. KDM6B inhibition has shown antitumour activity in preclinical cancer models, and KDM6‐family inhibitors such as GSK‐J4/GSK‐J1 support the feasibility of targeting this epigenetic regulator [31]. In addition, DKK1 blockade, exemplified by the anti‐DKK1 antibody DKN‐01, and Wnt/β‐catenin pathway modulators may provide complementary strategies [32, 33]. However, these approaches require further validation in CRC peritoneal metastasis models, particularly regarding therapeutic window, toxicity and patient selection.

Certainly, this study has certain limitations: first, the conclusions are primarily based on retrospective data and preclinical models, and the clinical predictive value of DKK1 requires further validation in prospective, multi‐centre cohorts; second, whether the role of DKK1 in peritoneal metastasis depends on the unique peritoneal microenvironment (such as mesothelial cells, peritoneal immune cells, ascites components, etc.) has not been thoroughly explored; furthermore, whether DKK1 influences tumour stem cell properties or chemotherapy resistance awaits further investigation.

## 5 Conclusion

In summary, this study systematically clarifies the key driver axis of colorectal cancer peritoneal metastasis—KDM6B–DKK1–Wnt/β‐catenin–EMT—providing a complete chain of evidence from molecular characteristics and functional mechanisms to epigenetic regulation. These findings not only deepen the understanding of the biological uniqueness of peritoneal metastasis but also lay an important theoretical foundation for developing precise diagnostic and targeted therapeutic strategies for this metastatic subtype with poor prognosis.

## Author Contributions

**Zhichao Zhai:** conceptualization, methodology, writing – original draft, investigation, data curation, resources. **Ming Li:** conceptualization, writing – review and editing, project administration, supervision. **Jiajia Chen:** investigation, validation, visualization. **Yong Yang:** formal analysis, software, data curation.

## Funding

The authors have nothing to report.

## Ethics Statement

The acquisition of human tissue samples involved in this study was approved by the Ethics Committee of Peking University Shougang Hospital (Approval No. IRBK‐2026‐010‐02), and written informed consent was obtained from all patients. Animal experiments were approved by the Animal Ethics Committee of Peking University (Approval No. LA2020404) and were conducted in strict accordance with guidelines for the welfare and ethical use of laboratory animals.

## Conflicts of Interest

The authors declare no conflicts of interest.

## Supporting information


**Table S1:** The colorectal cancer (CRC) cohort/datasets in this study.

**Table S2:** The primers used in the ChIP analysis.

**Table S3:** The Cox regression analysis of the DEGs.

**Table S4:** Gene set enrichment analysis (GSEA) between high and low DKK1 expression groups.

## Data Availability

Public transcriptomic data used in this study were obtained from The Cancer Genome Atlas (TCGA) database (https://portal.gdc.cancer.gov/) and the Gene Expression Omnibus (GEO) database (https://www.ncbi.nlm.nih.gov/geo/). Data generated during the experimental procedures are available from the corresponding author upon reasonable request.
