# Supplementary material for: Epigenetic Activation of the KDM6B‐DKK1 Axis Promotes Colorectal Cancer Peritoneal Metastasis Through Wnt/β‐Catenin‐Associated EMT
Source: J Cell Mol Med. 2026 Aug 23;30(16):e71326. doi: 10.1111/jcmm.71326 (PMC13500894; doi:10.1111/jcmm.71326)
Supplement: Supplementary file 1 [file JCMM-30-e71326-s001.docx]

**Table S4. Gene Set Enrichment Analysis (GSEA) between high and low DKK1 expression groups.**

| NAME | GS<br> follow link to MSigDB | GS DETAILS | SIZE | ES | NES | NOM p-val | FDR q-val | FWER p-val | RANK AT MAX | LEADING EDGE |
| --- | --- | --- | --- | --- | --- | --- | --- | --- | --- | --- |
| HALLMARK_HEME_METABOLISM | HALLMARK_HEME_METABOLISM | Details ... | 188 | 0.39564082 | 1.6446484 | 0 | 0.04700001 | 0 | 3850 | tags=26%, list=23%, signal=33% |
| HALLMARK_MYOGENESIS | HALLMARK_MYOGENESIS | Details ... | 196 | 0.6318014 | 1.5382732 | 0 | 0.20871428 | 0.139 | 2757 | tags=40%, list=16%, signal=48% |
| HALLMARK_APICAL_JUNCTION | HALLMARK_APICAL_JUNCTION | Details ... | 193 | 0.6311128 | 1.536818 | 0 | 0.15480946 | 0.139 | 2762 | tags=40%, list=16%, signal=48% |
| HALLMARK_COAGULATION | HALLMARK_COAGULATION | Details ... | 137 | 0.6627497 | 1.5231177 | 0 | 0.12785715 | 0.139 | 2466 | tags=43%, list=15%, signal=50% |
| HALLMARK_COMPLEMENT | HALLMARK_COMPLEMENT | Details ... | 198 | 0.65303224 | 1.4899927 | 0 | 0.14966898 | 0.192 | 2433 | tags=39%, list=14%, signal=45% |
| HALLMARK_HYPOXIA | HALLMARK_HYPOXIA | Details ... | 197 | 0.6293975 | 1.4782115 | 0 | 0.15262415 | 0.248 | 2791 | tags=40%, list=16%, signal=47% |
| HALLMARK_IL2_STAT5_SIGNALING | HALLMARK_IL2_STAT5_SIGNALING | Details ... | 196 | 0.5879146 | 1.4709363 | 0 | 0.1720885 | 0.293 | 3341 | tags=47%, list=20%, signal=58% |
| HALLMARK_ADIPOGENESIS | HALLMARK_ADIPOGENESIS | Details ... | 194 | 0.5370996 | 1.4593326 | 0 | 0.17737754 | 0.293 | 2223 | tags=22%, list=13%, signal=25% |
| HALLMARK_ESTROGEN_RESPONSE_EARLY | HALLMARK_ESTROGEN_RESPONSE_EARLY | Details ... | 194 | 0.4860236 | 1.4506938 | 0 | 0.16289113 | 0.293 | 2916 | tags=31%, list=17%, signal=37% |
| HALLMARK_APOPTOSIS | HALLMARK_APOPTOSIS | Details ... | 160 | 0.5985091 | 1.4486591 | 0 | 0.15627079 | 0.345 | 3321 | tags=41%, list=20%, signal=51% |
| HALLMARK_ANGIOGENESIS | HALLMARK_ANGIOGENESIS | Details ... | 35 | 0.779477 | 1.4368197 | 0 | 0.1573945 | 0.448 | 1714 | tags=57%, list=10%, signal=63% |
| HALLMARK_INFLAMMATORY_RESPONSE | HALLMARK_INFLAMMATORY_RESPONSE | Details ... | 200 | 0.6615916 | 1.4018579 | 0 | 0.15846705 | 0.448 | 3341 | tags=52%, list=20%, signal=64% |
| HALLMARK_REACTIVE_OXYGEN_SPECIES_PATHWAY | HALLMARK_REACTIVE_OXYGEN_SPECIES_PATHWAY | Details ... | 49 | 0.46348462 | 1.3960811 | 0 | 0.16653293 | 0.506 | 5460 | tags=47%, list=32%, signal=69% |
| HALLMARK_TNFA_SIGNALING_VIA_NFKB | HALLMARK_TNFA_SIGNALING_VIA_NFKB | Details ... | 194 | 0.7037355 | 1.3937562 | 0 | 0.15799469 | 0.506 | 3437 | tags=64%, list=20%, signal=80% |
| HALLMARK_UV_RESPONSE_UP | HALLMARK_UV_RESPONSE_UP | Details ... | 156 | 0.45037484 | 1.38826 | 0 | 0.15470421 | 0.506 | 3426 | tags=28%, list=20%, signal=35% |
| HALLMARK_P53_PATHWAY | HALLMARK_P53_PATHWAY | Details ... | 190 | 0.4556125 | 1.3853436 | 0.09288538 | 0.14797264 | 0.506 | 5105 | tags=47%, list=30%, signal=66% |
| HALLMARK_KRAS_SIGNALING_UP | HALLMARK_KRAS_SIGNALING_UP | Details ... | 196 | 0.6568982 | 1.3800246 | 0 | 0.14565843 | 0.506 | 3038 | tags=53%, list=18%, signal=63% |
| HALLMARK_APICAL_SURFACE | HALLMARK_APICAL_SURFACE | Details ... | 44 | 0.490123 | 1.3798673 | 0 | 0.1401775 | 0.506 | 2243 | tags=23%, list=13%, signal=26% |
| HALLMARK_EPITHELIAL_MESENCHYMAL_TRANSITION | HALLMARK_EPITHELIAL_MESENCHYMAL_TRANSITION | Details ... | 197 | 0.8240797 | 1.3737757 | 0 | 0.14170547 | 0.56 | 1730 | tags=70%, list=10%, signal=77% |
| HALLMARK_ESTROGEN_RESPONSE_LATE | HALLMARK_ESTROGEN_RESPONSE_LATE | Details ... | 195 | 0.4572323 | 1.3729634 | 0 | 0.13697013 | 0.56 | 2263 | tags=26%, list=13%, signal=29% |
| HALLMARK_UV_RESPONSE_DN | HALLMARK_UV_RESPONSE_DN | | 139 | 0.6913611 | 1.3692173 | 0 | 0.13268597 | 0.56 | 2788 | tags=51%, list=16%, signal=61% |
| HALLMARK_TGF_BETA_SIGNALING | HALLMARK_TGF_BETA_SIGNALING | | 53 | 0.6761863 | 1.3602868 | 0 | 0.12879105 | 0.56 | 3837 | tags=62%, list=23%, signal=80% |
| HALLMARK_XENOBIOTIC_METABOLISM | HALLMARK_XENOBIOTIC_METABOLISM | | 194 | 0.4093107 | 1.3592095 | 0 | 0.1252351 | 0.56 | 2390 | tags=23%, list=14%, signal=26% |
| HALLMARK_NOTCH_SIGNALING | HALLMARK_NOTCH_SIGNALING | | 32 | 0.57248694 | 1.3291148 | 0 | 0.13462807 | 0.56 | 4751 | tags=53%, list=28%, signal=74% |
| HALLMARK_ANDROGEN_RESPONSE | HALLMARK_ANDROGEN_RESPONSE | | 95 | 0.49911252 | 1.319058 | 0 | 0.13743773 | 0.594 | 3127 | tags=34%, list=18%, signal=41% |
| HALLMARK_ALLOGRAFT_REJECTION | HALLMARK_ALLOGRAFT_REJECTION | | 194 | 0.6041368 | 1.2501405 | 0.19726562 | 0.21974881 | 0.751 | 3695 | tags=49%, list=22%, signal=63% |
| HALLMARK_IL6_JAK_STAT3_SIGNALING | HALLMARK_IL6_JAK_STAT3_SIGNALING | | 87 | 0.6068019 | 1.2444009 | 0.08793456 | 0.2214073 | 0.751 | 2267 | tags=36%, list=13%, signal=41% |
| HALLMARK_BILE_ACID_METABOLISM | HALLMARK_BILE_ACID_METABOLISM | | 109 | 0.32751453 | 1.218156 | 0 | 0.26274702 | 0.79 | 3274 | tags=25%, list=19%, signal=30% |
| HALLMARK_INTERFERON_GAMMA_RESPONSE | HALLMARK_INTERFERON_GAMMA_RESPONSE | | 192 | 0.62027794 | 1.2176307 | 0.28627452 | 0.25530744 | 0.79 | 3024 | tags=41%, list=18%, signal=49% |
| HALLMARK_HEDGEHOG_SIGNALING | HALLMARK_HEDGEHOG_SIGNALING | | 36 | 0.4651158 | 1.2118205 | 0.06910569 | 0.2596967 | 0.79 | 2583 | tags=33%, list=15%, signal=39% |
| HALLMARK_PROTEIN_SECRETION | HALLMARK_PROTEIN_SECRETION | | 95 | 0.40786523 | 1.154068 | 0.19760479 | 0.32181323 | 0.848 | 5153 | tags=39%, list=30%, signal=56% |
| HALLMARK_GLYCOLYSIS | HALLMARK_GLYCOLYSIS | | 196 | 0.3848154 | 1.1539773 | 0.18762475 | 0.31322524 | 0.848 | 3679 | tags=27%, list=22%, signal=34% |
| HALLMARK_FATTY_ACID_METABOLISM | HALLMARK_FATTY_ACID_METABOLISM | | 151 | 0.37626845 | 1.146907 | 0.3013972 | 0.3093262 | 0.848 | 2333 | tags=19%, list=14%, signal=21% |
| HALLMARK_WNT_BETA_CATENIN_SIGNALING | HALLMARK_WNT_BETA_CATENIN_SIGNALING | | 41 | 0.4103944 | 1.0687566 | 0.4163424 | 0.43414766 | 0.951 | 3816 | tags=37%, list=22%, signal=47% |
| HALLMARK_UNFOLDED_PROTEIN_RESPONSE | HALLMARK_UNFOLDED_PROTEIN_RESPONSE | | 104 | 0.4439152 | 1.0603187 | 0.26347306 | 0.4331478 | 0.951 | 4966 | tags=43%, list=29%, signal=61% |
| HALLMARK_INTERFERON_ALPHA_RESPONSE | HALLMARK_INTERFERON_ALPHA_RESPONSE | | 92 | 0.50490415 | 0.986759 | 0.58235294 | 0.5808105 | 1 | 4303 | tags=41%, list=25%, signal=55% |
| HALLMARK_PI3K_AKT_MTOR_SIGNALING | HALLMARK_PI3K_AKT_MTOR_SIGNALING | | 104 | 0.29764557 | 0.96374446 | 0.4251497 | 0.6136961 | 1 | 5317 | tags=35%, list=31%, signal=50% |
| HALLMARK_KRAS_SIGNALING_DN | HALLMARK_KRAS_SIGNALING_DN | | 193 | 0.30384797 | 0.96318287 | 0.52286285 | 0.5987832 | 1 | 1397 | tags=11%, list=8%, signal=12% |
| HALLMARK_CHOLESTEROL_HOMEOSTASIS | HALLMARK_CHOLESTEROL_HOMEOSTASIS | | 71 | 0.4361637 | 0.95845413 | 0.46307385 | 0.58918643 | 1 | 4621 | tags=42%, list=27%, signal=58% |
| HALLMARK_MITOTIC_SPINDLE | HALLMARK_MITOTIC_SPINDLE | | 198 | 0.32714856 | 0.9026314 | 0.81237525 | 0.6513193 | 1 | 4290 | tags=33%, list=25%, signal=43% |
| HALLMARK_MTORC1_SIGNALING | HALLMARK_MTORC1_SIGNALING | | 191 | 0.39577636 | 0.9002517 | 0.6966068 | 0.63779163 | 1 | 4409 | tags=31%, list=26%, signal=42% |
| HALLMARK_DNA_REPAIR | HALLMARK_DNA_REPAIR | | 144 | 0.16639961 | 0.46875942 | 0.90618765 | 1 | 1 | 5754 | tags=31%, list=34%, signal=47% |
| HALLMARK_OXIDATIVE_PHOSPHORYLATION | HALLMARK_OXIDATIVE_PHOSPHORYLATION | | 181 | 0.1030524 | 0.25344855 | 0.90618765 | 1 | 1 | 4497 | tags=13%, list=27%, signal=18% |
