# Supplementary material for: Epigenetic Activation of the KDM6B‐DKK1 Axis Promotes Colorectal Cancer Peritoneal Metastasis Through Wnt/β‐Catenin‐Associated EMT
Source: J Cell Mol Med. 2026 Aug 23;30(16):e71326. doi: 10.1111/jcmm.71326 (PMC13500894; doi:10.1111/jcmm.71326)
Supplement: Supplementary file 2 [file JCMM-30-e71326-s002.docx]

**Table S3. The Cox regression analysis of the DEGs.**

| **Top 20** | **coef** | **se** | **z** | **p** | **HR** | **HRCILL** |
| --- | --- | --- | --- | --- | --- | --- |
| DNAH14 | 0.773 | 0.229 | 3.375 | 0.001 | 2.167 | 1.383 |
| FJX1 | 0.682 | 0.224 | 3.049 | 0.002 | 1.977 | 1.276 |
| PTF1A | 0.762 | 0.252 | 3.026 | 0.002 | 2.143 | 1.308 |
| **DKK1** | **0.710** | **0.229** | **3.097** | **0.002** | **2.033** | **1.298** |
| E2F1 | 0.674 | 0.224 | 3.004 | 0.003 | 1.963 | 1.264 |
| AURKA | 0.668 | 0.223 | 2.991 | 0.003 | 1.950 | 1.259 |
| SIX1 | 0.660 | 0.231 | 2.858 | 0.004 | 1.935 | 1.230 |
| IGF2BP1 | 0.630 | 0.224 | 2.805 | 0.005 | 1.877 | 1.209 |
| ISM2 | 0.604 | 0.221 | 2.729 | 0.006 | 1.829 | 1.185 |
| ARID3A | 0.586 | 0.227 | 2.578 | 0.010 | 1.797 | 1.151 |
| LRRC43 | 0.565 | 0.222 | 2.546 | 0.011 | 1.760 | 1.139 |
| MYCN | 0.571 | 0.224 | 2.547 | 0.011 | 1.770 | 1.141 |
| RSPO4 | -0.564 | 0.222 | -2.539 | 0.011 | 0.569 | 0.368 |
| NKX2-1 | 0.620 | 0.248 | 2.498 | 0.012 | 1.858 | 1.143 |
| SERPINE2 | 0.565 | 0.224 | 2.518 | 0.012 | 1.760 | 1.133 |
| HOXC6 | -0.556 | 0.224 | -2.480 | 0.013 | 0.574 | 0.370 |
| B4GALNT4 | 0.547 | 0.220 | 2.483 | 0.013 | 1.728 | 1.122 |
| E2F5 | 0.542 | 0.221 | 2.452 | 0.014 | 1.719 | 1.115 |
| FGA | 0.547 | 0.222 | 2.467 | 0.014 | 1.728 | 1.119 |
| TRIM29 | -0.550 | 0.226 | -2.433 | 0.015 | 0.577 | 0.371 |
