# Supplementary material for: Epigenetic Activation of the KDM6B‐DKK1 Axis Promotes Colorectal Cancer Peritoneal Metastasis Through Wnt/β‐Catenin‐Associated EMT
Source: J Cell Mol Med. 2026 Aug 23;30(16):e71326. doi: 10.1111/jcmm.71326 (PMC13500894; doi:10.1111/jcmm.71326)
Supplement: Supplementary file 3 [file JCMM-30-e71326-s003.docx]

**Table S1. The colorectal cancer (CRC) cohort/datasets in this study.**

| Datasets | Normal | Primary | Peritoneum (PM) | Liver | Lung | Lymph nodes |
| --- | --- | --- | --- | --- | --- | --- |
| TCGA | 101 | 624 |  |  |  |  |
| GSE225182 | 7 | 19 | 7 |  |  |  |
| GSE147971 |  | 3 | 14 |  |  |  |
| GSE41568 |  | 39 | 5 | 80 | 8 |  |
| GSE68468 | 75 | 195 |  | 47 | 20 |  |
| GSE26571 | 5 | 12 |  |  |  | 9 |
| GSE3964 | 6 | 9 |  | 14 |  |  |
| GSE6988 | 42 | 53 |  | 13 |  |  |
| GSE62321 | 18 | 20 |  | 19 |  |  |
