# Supplementary material for: Epigenetic Activation of the KDM6B‐DKK1 Axis Promotes Colorectal Cancer Peritoneal Metastasis Through Wnt/β‐Catenin‐Associated EMT
Source: J Cell Mol Med. 2026 Aug 23;30(16):e71326. doi: 10.1111/jcmm.71326 (PMC13500894; doi:10.1111/jcmm.71326)
Supplement: Supplementary file 4 [file JCMM-30-e71326-s004.docx]

**Table S2. The primers used in the ChIP analysis.**

| chrom | start_0based | end_halfopen | name | score | strand | relative_window |
| --- | --- | --- | --- | --- | --- | --- |
| chr10 | 52313230 | 52313387 | P1 | 0 | + | -1050..-894 |
| chr10 | 52313387 | 52313544 | P2 | 0 | + | -893..-737 |
| chr10 | 52313544 | 52313701 | P3 | 0 | + | -736..-580 |
| chr10 | 52313701 | 52313858 | P4 | 0 | + | -579..-423 |
| chr10 | 52313858 | 52314015 | P5 | 0 | + | -422..-266 |
| chr10 | 52314015 | 52314171 | P6 | 0 | + | -265..-110 |
| chr10 | 52314160 | 52314361 | P7 | 0 | + | -120..80 |
| chr10 | 52314325 | 52314481 | P8 | 0 | + | 45..200 |
